# Numerical investigation of laterally loaded pile groups at the crest of slopes

**DOI:** 10.1038/s41598-025-07715-x

**Published:** 2025-07-02

**Authors:** Meen-Wah Gui, Alex A. Alebachew

**Affiliations:** Department of Civil Engineering, National Taipei University of Technology (Taipei Tech), 106344 Taipei, Taiwan

**Keywords:** Lateral load, Pile group, Slope, Numerical analysis, Shear resistance, Pile spacing, Civil engineering, Engineering

## Abstract

Pile group foundations are widely used to support tall structures on both level and sloping terrain. However, predicting the lateral response of pile groups near slopes remains challenging due to complex soil-pile interactions that differ significantly from those on level ground. This study employed 3D finite element (FE) analysis using COMSOL v5.6 to investigate the behavior of laterally loaded $2 \times 2$ pile groups positioned on the crest of slopes. Key variables included pile spacing (*s*/*D* = 3 to 6), slope gradient (1:1 to 1:2.5), and setback distance from the slope crest (*b*/*D* = 0 to 12). The results revealed critical insights. A setback ratio $b/D \le 12$ reduced lateral capacity by 15–35% compared to level ground, establishing $b/D=12$ as a critical threshold for design. Steep slopes (1:1) degraded lateral capacity by 5–20% compared to gentle slopes (1:2.5), highlighting the sensitivity of pile performance to slope steepness. Wider spacing (*s*/*D* = 6) improved lateral resistance by 40–50% over tightly spaced groups (*s*/*D* = 3) due to reduced group interaction effects. The lateral capacity of pile groups near slopes was governed by the sliding resistance of the passive soil wedge adjacent to the slope, analogous to a stabilizing berm in retaining wall design. The findings emphasize that setback distance is the most influential design parameter, followed by slope gradient and pile spacing. This study provides actionable guidelines for optimizing pile group configurations near slopes, ensuring safer and more economical designs.

## Introduction

Pile group foundations are indispensable for supporting tall structures on both level and sloping terrain. While piles exhibit greater flexibility in bending compared to axial loading, their lateral behavior–especially for single piles or groups near slopes–is challenging to predict using conventional analysis due to complex soil-pile interactions. Unlike piles on level ground, those near slopes exhibit distinct interaction mechanisms influenced by slope geometry, pile spacing, and arrangement^1^. For instance, closer spacing amplifies group interaction effects, altering the system’s lateral response. Increasing pile spacing reduces these interactions, allowing individual piles to behave more independently; however, this may compromise the group’s collective lateral capacity^2^. The unique challenges of sloping terrains underscore the importance of studying pile behavior near slopes. Research on single piles adjacent to slopes highlights critical parameters such as slope gradient, setback distance *b* from the slope crest, and soil properties^3^. These factors collectively govern the stability and performance of pile-supported structures in such environments.

Sivapriya and Ramanathan^4^ experimentally studied the lateral load capacity of single piles on sloping ground under varying slope gradients, pile lengths, and pile diameters. Their findings revealed lateral load capacity diminishes as slope gradients increase, primarily due to reduced passive soil resistance. Additionally, lateral capacity improves when the pile is loaded away from the slope crest, mirroring the behavior of piles on horizontal ground. Additionally, longer piles and those with larger diameters exhibited higher lateral load capacity. The study also observed that the elastic deflection of the pile increases with steeper slope gradients.

Muthukkumaran and Begum^5^ conducted laboratory model tests to investigate the effects of ground slope, soil density, and pile embedment length on the lateral load capacity of single piles in sloping terrain. Their study revealed that higher soil density and greater pile embedment length enhance lateral resistance, whereas steeper ground slopes reduce it. Similar to Sivapriya and Ramanathan^4^, the research did not address pile spacing or group configurations, as it focused exclusively on single piles.

Experimental and numerical studies on pile groups remain relatively limited. Khati and Sawant^6^ conducted experimental studies on the lateral response of 2-pile groups (series and parallel) positioned on sloping terrain composed of dry Solani river sand. Their findings indicated that lateral pile capacity decreases while bending moments increase, with reduced edge distance of the pile group. In their subsequent study^7^, Khati and Sawant investigated the lateral behavior of a $2 \times 2$ pile group on sloping ground (dry Solani river sand) by varying the relative density, slope gradient, and edge distance. Results demonstrated significant reductions in pile capacity near slopes, particularly at the crest, with maximum decreases ranging from 41.6% to 51.5% depending on relative density. Additionally, bending moments increased by up to 102.9% in loose sand.

Recent advancements in numerical modeling have enhanced the understanding of pile behavior near sloping ground. Abbas et al.^8^ employed the ABAQUS finite element method to analyze single piles and $2 \times 2$ pile groups under lateral loads in the sand, investigating the effects of pile spacing on *p*-multipliers and group efficiency. Their analysis revealed that *p*-multipliers are higher for leading piles than trailing piles at spacings up to five times the pile diameter (5*D*), approaching unity at 6*D*. In contrast, group efficiency increased with spacing, achieving nearly 100% at 7*D*.

Bahloul^9^ utilized PLAXIS 3D to analyze the response of laterally loaded single piles, $2 \times 1$, and $2 \times 3$ pile groups on level ground and a 2*V* : 3*H* slope. The study found that pile groups exhibit similar lateral capacity on level ground, regardless of their arrangement. However, the slope’s influence on lateral capacity is more pronounced in parallel configurations (lower second-moment-of-inertia) compared to series configurations (higher second-moment-of-inertia). The slope effect diminishes when the setback distance *B* exceeds 5*D* for series arrangements and 8*D* for parallel arrangements. These findings, however, are specific to loose sand and the 2*V* : 3*H* slope geometry.

Sivapriya and Gandhi^10^ performed 1*g* laboratory experiments and PLAXIS 3D numerical analyses to evaluate the lateral capacity of single piles and $2 \times 1$ pile groups on clayey sloping ground. The study examined the effects of soil shear strength, slope gradient, and pile location variations. Results revealed that lateral capacity decreases by up to 42% with steeper slope gradients and larger setback distances from the slope crest.

Deendayal et al.^1^ examined the performance of $3 \times 3$ pile groups on sloping ground surfaces (1*V* : 5*H* and 1*V* : 3*H* slopes) in soft to medium-stiff clay and silts underlain by sand. Their study highlighted the influence of slope geometry on pile deflections and bending moments, with the front row piles exhibiting the maximum displacement at the pile head, followed by the middle and back rows. Alinejad et al.^11^ experimentally investigated the response of a $2 \times 2$ pile group near the crest of a slope under axial loading in poorly graded sand. They found that greater embedded lengths produced responses similar to pile groups on level ground. Navale et al.^12^ employed three-dimensional (3D) nonlinear finite element analysis to study $2 \times 2$ pile groups near sloping ground. Their results showed that the point of zero-shear (where maximum bending moment occurs) shifts deeper along the pile’s depth as the slope gradient increases. These studies highlight the necessity of integrating slope-specific parameters to understand better the behavior of laterally loaded pile groups near sloping terrain.

Research on pile groups at the crest of sloping ground remains limited. Deendayal et al.^1^ emphasize a critical gap in understanding pile group behavior near slopes due to insufficient experimental data. While laboratory 1*g* experiments struggle to replicate in-situ stress-dependent soil properties, full-scale field tests are time-consuming, costly, and prone to technical challenges. However, advances in computational hardware and numerical tools enable simulations to address these experimental limitations. 3D finite element (FE) modeling is preferable to 2D analyses for studying pile group behavior near slopes, as the latter fails to capture pile-soil interactions in complex geometries fully^13^.

Existing research on laterally loaded piles has predominantly focused on single piles or simplified configurations, neglecting the complex three-dimensional interactions inherent to pile groups near slope crests. While prior investigations have advanced understanding through small-scale experiments and 2D numerical analyses, these approaches inadequately capture the full spatial soil-pile interaction mechanisms, particularly for cohesive-frictional ($c'-\phi '$) soils – a prevalent yet understudied material type in practice, as most studies restrict analysis to purely cohesive or cohesionless soils. Critical design parameters – pile spacing ratio (*s*/*D*), setback ratio (*b*/*D*), and slope gradient (1 : *n*) – have not been systematically examined in tandem despite their demonstrated individual influence on lateral load response. This oversight limits comprehensive insights into how these interdependent variables collectively govern pile group performance, especially in slopes composed of $c'-\phi '$ soils where combined shear strength mechanisms dictate behavior. The absence of integrated parametric analysis leaves a gap in practical frameworks for optimizing slope-adjacent pile group designs under lateral loading.

To address these gaps, this study investigated the behavior of laterally loaded 2$\times$2 pile groups installed at varying spacings and setback distances from the crest of slopes with different gradients. The 2$\times$2 pile group configuration was selected to minimize edge effects (overlapping zones of influence between piles in the same row) and shadowing effects (overlapping between piles in different rows), both of which reduced lateral resistance per pile due to group interaction^14,15^. By addressing these factors this research helps us better understand how pile groups behave when placed near slopes. It also offers practical tips to improve how engineers design these foundations in real-world situations.

## Materials and methods

**Figure 1:**
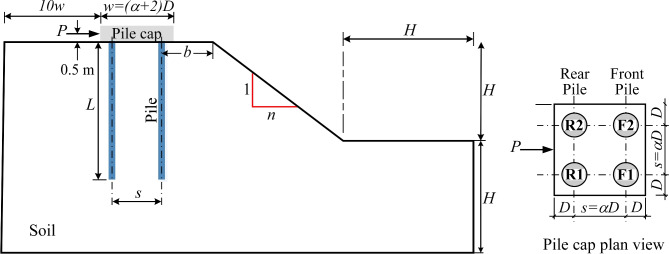
A schematic layout depicts the simulated group pile located near the crest of a slope.

This study adopts a $2 \times 2$ pile group layout, initially used in Khati and Sawant^7^ but with modified dimensions, positioned near the crest of a homogeneous porous soil slope (Fig. 1). The piles were subjected to lateral loading through a square concrete pile cap of width $w=(\alpha +2)D$ m, where $\alpha$ $(=s/D)$ represents the ratio of pile spacing *s* to diameter *D*. The pile cap rested on the ground surface, with piles measuring 0.5 m in diameter and 15 m in length (*L*). As the pile diameter was less than 0.55 m, the cap thickness was set to 2D, consistent with McCormac and Brown^16^, who state that this thickness ensures effective transfer of horizontal loads from the cap to the pile group.

This study examines the behavior of isolated pile groups under lateral loading, with the pile cap modeled as a rigid element to ensure effective load redistribution. Shear resistance at the cap–soil interface is deliberately excluded to isolate the effects of soil–pile interaction, which primarily governs lateral resistance through mechanisms like passive pressure along the pile shafts and toes. Previous research on piled raft systems indicates that the raft’s role in lateral resistance is typically minor, contributing less than 5% variation in pile forces^17,18^. Mandolini et al.^19^ also observed that the raft’s overall contribution was limited in small piled rafts (raft width/pile length < 1), while Jamili et al.^20^ found the raft’s lateral resistance to be proportional to vertical pressure. Since no vertical loads were applied in this study, the raft’s influence is negligible. Although raft–soil friction might play a role in shallow or soft soil conditions, its effect is minimal under the conditions analyzed here, with lateral displacements up to 100 mm. Future studies may revisit this assumption in the more complex raft–pile–soil interaction models.

To model soil behavior with high fidelity, Li et al.^21^ numerically investigated the lateral response of $3 \times 3$ pile groups in level Toyoura sand using an advanced hypoplastic model. This model requires 13 parameters – significantly more than the Mohr-Coulomb model – but introduces greater uncertainty in parameter determination, potentially affecting result reliability. In contrast, Khati and Sawant^7^ employed a purely frictional soil model.

This study advances prior approaches by adopting a $c'-\phi '$ soil model, incorporating both apparent cohesion $c'$ and friction angle $\phi '$, to better represent diverse soil behaviors, including cohesive effects. Transitioning from a purely frictional to a $c'-\phi '$ framework enables a more realistic simulation of soil under lateral loading.

The concrete pile group and cap were modeled as linear elastic materials. For the porous soil slope, a Mohr-Coulomb-matched Drucker-Prager elastoplastic model was applied, combining the Mohr-Coulomb model’s $c'$ and $\phi '$ with the Drucker-Prager model’s smooth elastic-plastic transition^22^. This hybrid approach captures nonlinear elastoplastic soil behavior under stress. Table 1 summarizes the input parameters.

**Table 1:** Materials property used in numerical analysis.

Materials property	Value
Pile length, *L*	15 m
Pile diameter, *D*	0.5 m
Width of pile cap, *w*	$(\alpha +2)D$ m
Thickness of pile cap	1.0 m
Poisson’s ratio of concrete	0.2
Unit weight of concrete	25 kN/$\hbox {m}^3$
Young’s modulus of concrete pile, $E_p$	30 GPa
Unit weight of soil, $\gamma$	20 kN/$\hbox {m}^3$
Young’s modulus of soil	200 MPa
Porosity of soil	0.5
Hydraulic conductivity of soil	2.94 $\times 10^{-9}$ m/s
Poisson’s ratio of soil	0.3
Apparent cohesion, $c'$	50 kPa
Angle of internal friction, $\phi '$	30$^o$

**Figure 2:**
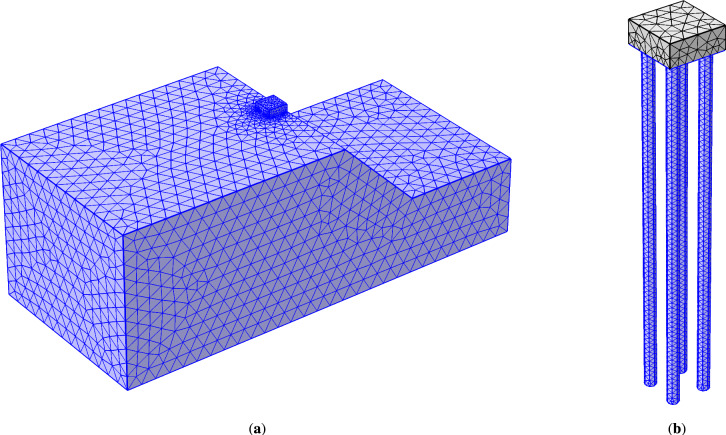
Finite element meshes generated using COMSOL^22^ for (**a**) the slope model; and (**b**) the pile group with a pile cap.

This study employs three-dimensional finite element analysis using COMSOL Multiphysics® (version 5.6, 2021)^22^ to simulate the mechanical behavior of the soil–pile–pile cap system. Figure 2 illustrates the finite element mesh of the soil slope and pile group. A graded mesh was implemented, with finer elements near the pile group and coarser elements toward the soil boundaries. For the pile group analysis, linear tetrahedral solid elements were selected, as^22^ recommends this discretization for quasi-static coupled analyses to enhance degree-of-freedom continuity across the model.

The governing equations were solved under time-independent equilibrium conditions, assuming progressive deformation without dynamic effects. A representative mesh for the 1*V* : 1.5*H* slope model included 105,505 domain elements, 6716 boundary elements, and 1376 edge elements, with variations in parametric studies for different slopes. The finalized geometry comprised six domains, 40 boundaries, 85 edges, and 56 vertices, reflecting the structural configuration.

To simulate the wish-in-place pile installation, the form union boundary operation in COMSOL was employed to merge adjacent boundaries between paired objects, i.e., pile-soil, ensuring fully bonded (no-slip) connectivity. This approach enforces continuity of displacement and stress across interfaces by maintaining non-sliding, conforming meshes along connected interior boundaries. Mesh refinement was strategically applied to regions of high-stress gradients, particularly near the pile-soil interfaces, to enhance numerical accuracy. While bonded contact assumptions align with the current study’s objectives, future work could explore incorporating contact or interface elements to model potential slip or separation at these boundaries. For methodological details, see^22^. The mesh achieved an average quality of 0.66 and a minimum of 0.19, exceeding the 0.1 threshold for acceptable meshing quality^22,23^. An implicit solver ensured numerical stability and convergence across all slope gradients.

The bottom boundary of the mesh was fixed in both vertical and horizontal directions. Conversely, the top boundary and slope surface remained unconstrained in all directions. All four vertical boundaries were restricted horizontally using roller supports. The groundwater table was aligned with the full height of the model’s left and right boundaries, and the analysis was conducted under fixed-head conditions.

This study employs a conventional effective stress analysis to evaluate soil-pile interactions under drained conditions. The analysis first calculated hydrostatic pore-water pressure and then activated the $2\times 2$ wish-in-place elastic pile group and pile cap at the slope crest to establish the model’s initial effective stress by gravity loading. Initial stresses in the soil and hydrostatic pressures from this stage were transferred to the subsequent analysis stage. Here, a lateral force *P* = 15 MN was applied incrementally to the pile cap’s left face, simulating a push toward the slope side. This lateral load was performed as a quasi-static simulation, with the load applied in 100 incremental steps to ensure numerical stability and capture the nonlinear response of the pile-soil system. Strain values from the prior stage were reset to zero to isolate incremental displacement caused solely by the lateral loading.

Numerous methods have been developed to analyze the response of laterally loaded piles, with particular emphasis on bending moment behavior. Among these, the Winkler spring model remains the most widely used approach. Winkler^24^ pioneered this method by modeling soil as a series of independent linear-elastic springs (termed Winkler springs), where the soil reaction at any point is proportional to the pile’s lateral deflection. Under this assumption, the lateral behavior of a pile is governed by a fourth-order differential equation for the elastic curve under zero axial load, representing a “special case” of the general formulation^25^:

1 $$\begin{aligned} E_pI_p\frac{d^4y}{dz^4} + ky = 0 \end{aligned}$$

where $E_p$ is the modulus of elasticity of the pile [F/$\hbox {L}^2$]; $I_p (=\frac{1}{4}\pi D^4)$ denotes the second-moment-of-inertia of the circular pile [$\hbox {L}^4$]; *y*(*z*) represents the lateral deflection of the pile at depth *z* (along the pile length) [L]; and *k* is the modulus of subgrade reaction (or soil stiffness per unit pile length) [F/L$^2$], defined as the ratio of soil reaction pressure to lateral deflection per unit length. According to Murthy^25^, the modulus of subgrade reaction *k* is central to resolving laterally loaded pile problems.

The Winkler spring method is applicable only for linear elastic soil behavior, whereas natural soils exhibit highly nonlinear, inelastic responses. To address this limitation, the linear springs in the Winkler model were replaced with nonlinear springs whose stiffness varies with pile deflection. This adaptation led to the $p-y$ curve method, where soil resistance *p* near the pile is represented either by a nonlinear function of deflection *y*(*z*)^26^ or by a fifth-degree polynomial fitted to experimental data^7^:

2 $$\begin{aligned} y(z) = \frac{1}{E_pI_p}\left( a_0 + a_1z + a_2z^2 +a_3z^3+ a_4z^4+a_4z^5\right) \end{aligned}$$

The bending moment at any depth *z*, *M*(*z*), can be derived using Euler-Bernoulli beam theory^27^. This theory relates *M*(*z*) to the pile’s flexural rigidity $E_pI_p$ and the second derivative of the pile deflection *y*(*z*) with respect to *z*:

3 $$\begin{aligned} M(z) = E_pI_p\frac{d^2y}{dz^2} \end{aligned}$$

## Results

This study investigated the influence of setback distance *b* from the slope crest (Fig. 2), pile spacing *s*, and slope gradient 1 : *n* on the lateral response of a pile group subjected to a 15 MN lateral load applied 0.5 m above the ground surface at the pile head level. Parameters included four normalized setback distances ($b/D=0,4,8,12$), three normalized pile spacings ($s/D=3,4,6$), and slope gradients (1*V* : *nH*) ranging from 1:1.0 to 1:2.5, as well as flat ground. A total of 51 finite element analyses were conducted: 48 on sloping ground and 3 on flat ground. The study analyzed the pile group’s response in terms of lateral deflection and bending moment distribution. Additionally, it assessed the combined effects of setback (*b*/*D*) and spacing (*s*/*D*) ratios on pile head displacement and lateral capacity.

### Lateral load–pile head displacement profile

**Figure 3:**
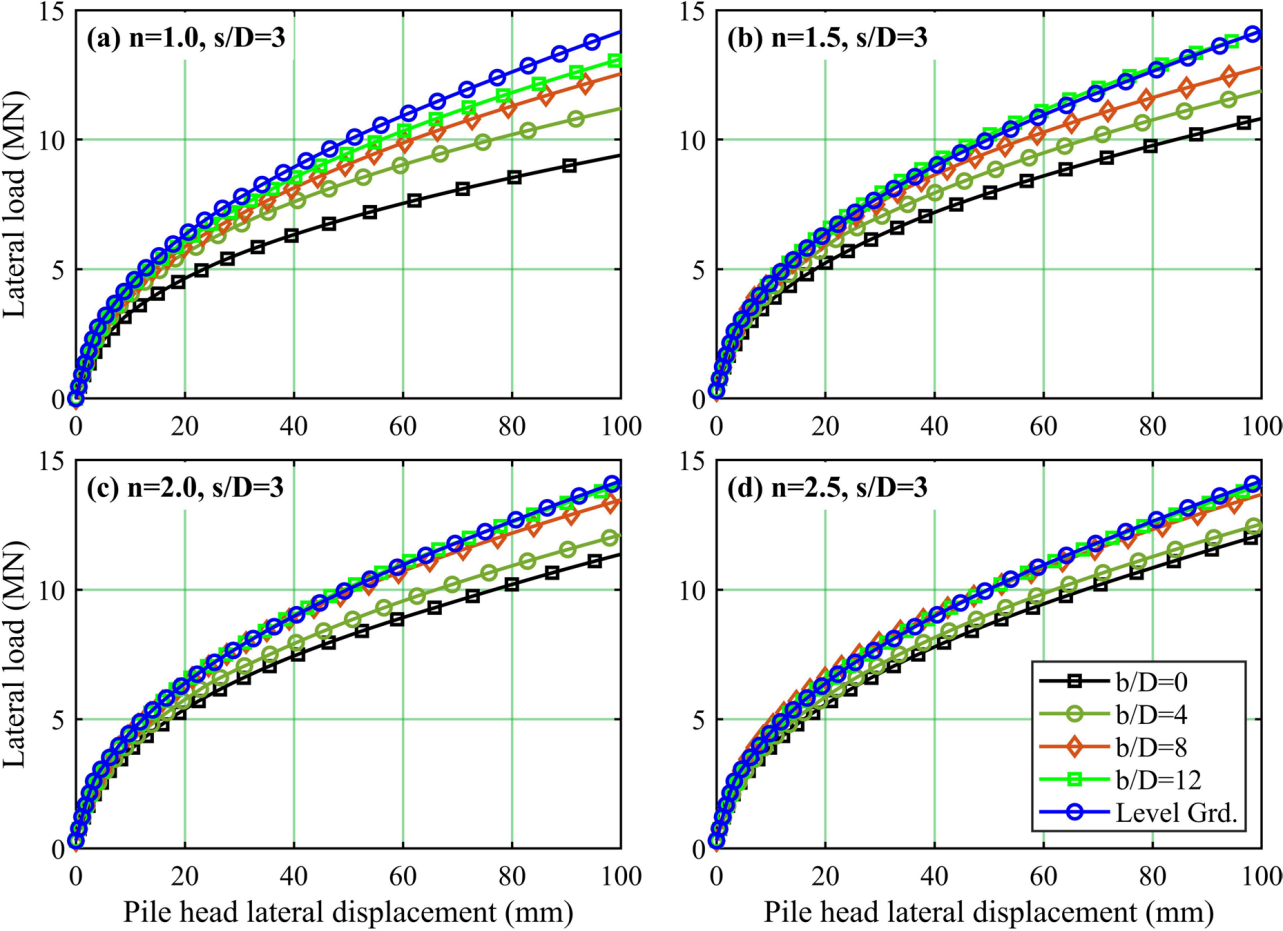
Typical relationship between lateral load and pile head lateral displacement in a 1*V* : *nH* slope: (**a**) $n=1.0$; (**b**) $n=1.5$; (**c**) $n=2.0$; (**d**) $n=2.5$.

Figure 3 illustrates the relationship between lateral load and lateral displacement for the front pile $F_1$. This pile is selected for analysis because it sustains the highest load within laterally loaded pile groups, as demonstrated by Elhakim et al.^2^, Ruesta and Townsend^28^, Rollins et al.^29^, and Rollins et al.^30^. In addition, studies^14,15^ have indicated that the shadowing effect may influence group effects and pile behavior. However, a key feature of sloped pile configurations is the uneven interaction between the soil and piles. The front pile, positioned near the top of the slope, experiences reduced passive soil resistance on its downslope side due to limited soil confinement. This reduction in resistance results in greater lateral movement, forcing the pile to rely more heavily on the soil’s remaining strength to resist the applied loads. In contrast, the rear pile, located further away from the slope face, is affected by shadowing caused by stress redistribution and soil already sheared from the front pile’s displacement. These conditions reduce the rear pile’s ability to mobilize undisturbed passive resistance. Although the slope’s asymmetric shape links the behavior of both piles, the front pile is significantly more vulnerable to destabilization from the slope. Therefore, the analysis concentrates on the response of front pile $F_1$, as it largely determines the failure mechanism of the system. While shadowing effects on the rear pile are acknowledged, they are excluded from the parametric study to keep the focus on the main factors driving slope-pile interaction.

Figure 3a–d present the load–pile head displacement profiles for different setback ratios (*b*/*D*) and slope gradients (1 : *n*); the piles in the group are spaced at 3*D* center-to-center. For comparison, each sub-figure also includes the corresponding profile for the level ground condition.

The analysis of lateral load versus pile head displacement reveals that the initial 100 mm displacement range for all slope gradients exhibits a nonlinear increase in lateral load. Note that the 100 mm lateral displacement adopted in this study does not correspond to a serviceability limit state. However, it acts as a controlled reference value to systematically compare parameter influences and estimate lateral load capacity. This displacement magnitude was standardized across parametric analyses to establish a consistent measurement baseline, facilitating direct comparison of pile behavior under significant lateral loading conditions.

For a given slope gradient (1 : *n*), higher setback ratios (*b*/*D*) require greater lateral loads to produce equivalent displacements. This behavior aligns with established pile response patterns, where increased *b*/*D* values demand higher lateral loading to induce comparable displacements, consistent with observations by Elhakim^2^. Notably, the $b/D=12$ configuration achieves the highest lateral load capacity, demonstrating that pile groups with larger setback ratios enhance lateral displacement resistance. These results highlight the critical influence of setback ratios in governing pile performance under lateral loading conditions.

The influence of the *b*/*D* setback ratio is most pronounced for the 1 : 1 slope gradient, where larger *b*/*D* values exhibit greater resistance to lateral deflection. The steep 1 : 1 slope develops the lowest lateral loads, increasing pile deflection under comparable loading conditions. As the slope gradient flattens from 1 : 1 to 1 : 2.5, the lateral load required to achieve equivalent deflection increases for identical *b*/*D* ratios. This behavior causes lateral load values to approach those measured on level ground, with convergence particularly evident for $b/D=12$ in the 1 : 1.5 slope and $b/D=8$ in the 1 : 2 slope gradient.

The steepest 1:1 slope exhibits the lowest lateral loads across all setback ratios (*b*/*D*), while the gentlest 1:2.5 slope demonstrates the highest. The disparity between level ground and $b/D=0$ load-displacement profiles is the greatest for the 1 : 1 slope, progressively decreasing as slopes transition to gentler gradients (1 : 1.5, 1 : 2, 1 : 2.5). These results indicate that reduced *b*/*D* ratios impair lateral load capacity more severely in steep slopes. This detrimental effect diminishes with flatter slopes, revealing a critical interdependence between slope inclination and the efficacy of setback ratios in governing pile response to lateral loads. Notably, lateral load values approach level-ground conditions for $b/D=12$ in the 1 : 1.5 slope and $b/D=8$ in the 1 : 2 slope, underscoring how slope geometry modulates setback ratio effectiveness.

### Pile lateral deflection profile

**Figure 4:**
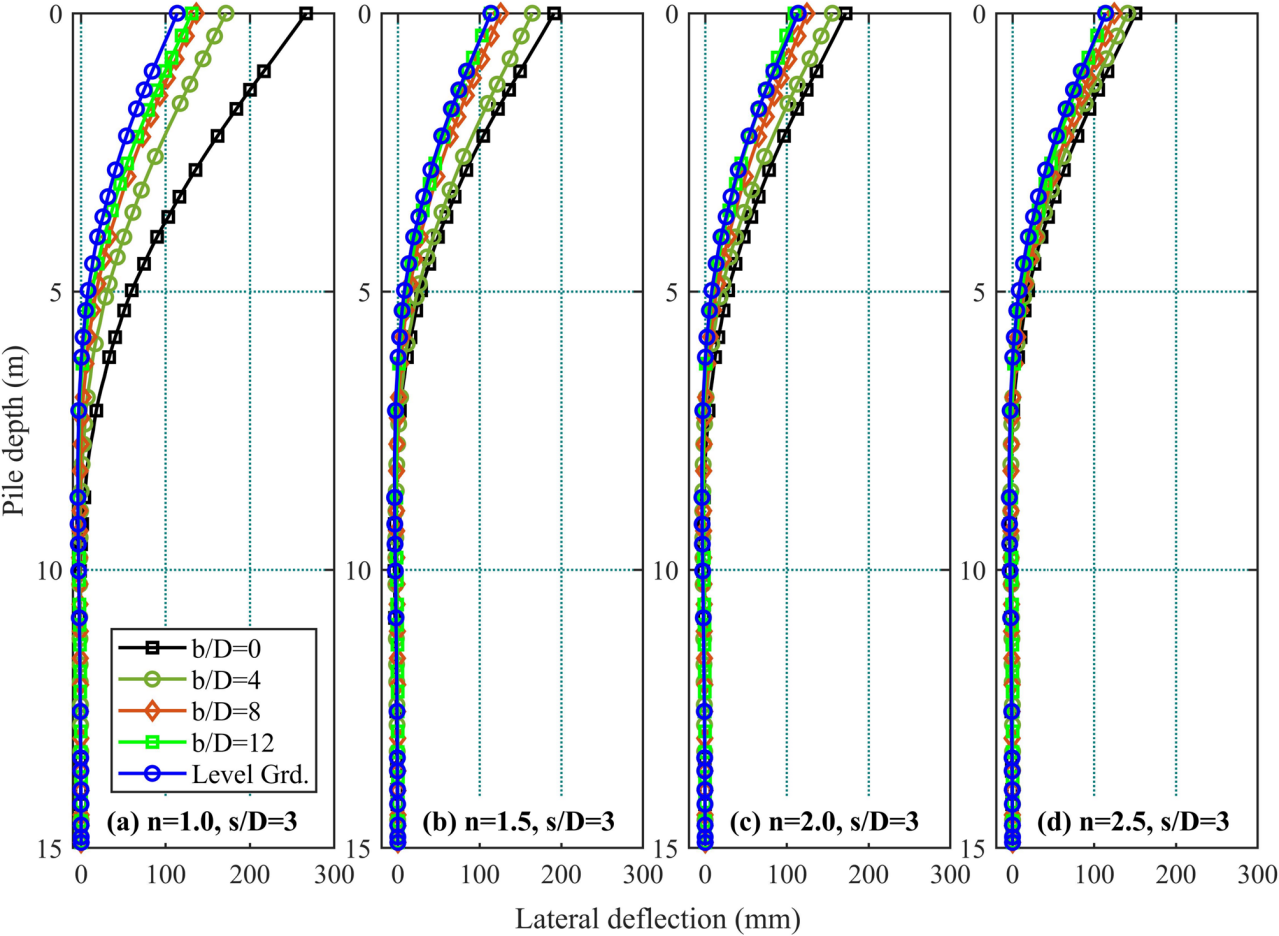
Pile lateral deflection profiles induced under different *b*/*D* conditions in a 1*V* : *nH* slope: (**a**) $=1.0$; (**b**) $n=1.5$; (**c**) $n=2.0$; and (**d**) $n=2.5$.

The study further examined lateral deflection patterns along front pile $F_1$ under a 15 MN lateral load, as depicted in Fig. 4. Figure 4a displays deflection profiles for different *b*/*D* ratios in the 1 : 1 slope, demonstrating that lateral deflection intensifies as the *b*/*D* ratio decreases. This confirms that smaller *b*/*D* ratios exacerbate lateral deflection in steep slopes, highlighting the ratio’s critical role in governing pile behavior under lateral forces. Across all slope gradients, higher *b*/*D* ratios consistently correlate with reduced lateral deflections.

Increasing the *b*/*D* ratio (positioning piles farther from the slope) mobilizes a greater soil mass ahead of the front piles, enhancing passive resistance. Conversely, near the slope crest (low *b*/*D*), the restricted soil volume diminishes lateral support. Relocating piles into level ground improves soil engagement, thereby reducing displacement. These mechanisms collectively demonstrate that larger *b*/*D* ratios enhance the serviceability of pile groups under lateral loading.

Deflection profiles show significant variation for $b/D \le 4$, particularly in slopes steeper than 1 : 2.0 (where $n \le 2.0$). This highlights how slope gradient critically influences soil-pile interaction and lateral load transfer mechanisms. In the 1 : 2.5 slope, all deflection profiles converge to within 25% of the $b/D=0$ case. Level ground conditions produce the smallest lateral deflections, confirming that steeper slopes amplify lateral displacements at equivalent pile depths. The pronounced deviation between steep slopes ($n \le 2.0$) and level ground diminishes as slopes flatten, with $b/D=0$ profiles in gentler gradients (e.g., 1 : 2.5) approaching level-ground behavior. These trends reinforce the dual dependence of pile deflection on both setback ratios and slope geometry.

### Combined effect of *b*/*D* and *s*/*D* on pile head lateral displacement

**Figure 5:**
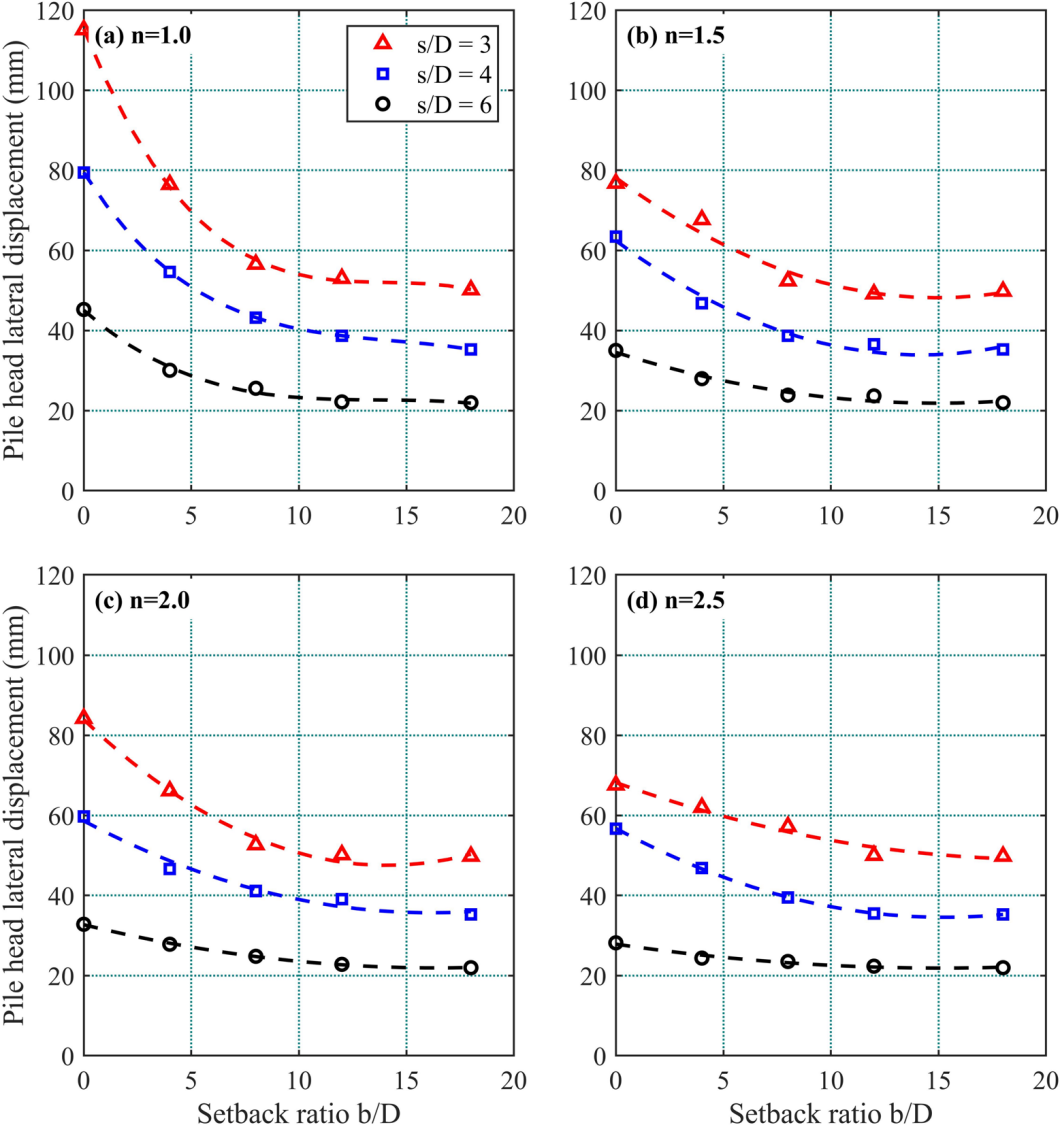
Effect of *b*/*D* and *s*/*D* on pile head lateral displacement under a 10 MN lateral load in a 1*V* : *nH* slope: (**a**) $=1.0$; (**b**) $n=1.5$; (**c**) $n=2.0$; and (**d**) $n=2.5$.

Figure 5 presents the lateral displacement at the pile head of front pile $F_1$ under a 10 MN lateral load, analyzed across different setback ratios (*b*/*D*), pile spacing ratios (*s*/*D*), and slope gradients (1 : *n*). For all tested pile spacing ratios (*s*/*D*), lateral displacement systematically decreases with increasing setback ratio (*b*/*D*). The data shows that *b*/*D* is inversely correlated with the displacement magnitude, indicating improved pile group stability with greater setback distances. The steady decrease in lateral displacement at higher *b*/*D* ratios highlights the parameter’s importance in enhancing lateral load resistance for different slope configurations.

Analysis of pile head lateral displacement across pile spacing ratios (*s*/*D*) at fixed setback ratios (*b*/*D*) reveals systematic trends. The $s/D=6$ configuration produces the lowest displacements for all slope conditions, followed by $s/D=4$, while $s/D=3$ yields the highest. Specifically, displacements for $s/D=4$ and $s/D=6$ average 72% and 42%, respectively, of those observed for $s/D=3$. These results demonstrate that increasing *b*/*D* and *s*/*D* enhances lateral resistance.

In the steep 1 : 1 slope ($n=1.0$), lateral displacements peak at 115 mm for $s/D=3$ with minimal *b*/*D* values. As *b*/*D* increases from 0 to 10, all *s*/*D* profiles show rapid displacement reduction, with the most pronounced improvements occurring within this range. Beyond $b/D=10$, displacement rates stabilize, indicating diminishing returns from further increases in setback distance. This behavior underscores the combined influence of slope gradient, pile spacing, and setback ratio on deformation control.

The lateral displacement trends for the $n=1.5$ slope mirror those of the steeper $n=1.0$ case but with marginally lower displacement magnitudes under identical *b*/*D* and *s*/*D* conditions. Displacement decreases sharply until $b/D=10$, after which the reduction rate diminishes. For gentler slopes ($n=2.0$ and $n=2.5$), lateral displacements decline universally across all *b*/*D* ratios, with the $s/D=6$ configuration exhibiting the most pronounced improvements. This confirms that flatter slopes (higher *n* values) inherently reduce lateral displacement.

Displacement reduction follows a consistent pattern: the steepest decreases occur at lower *b*/*D* values ($b/D<10$), while larger setbacks ($b/D \ge 10$) yield stabilized displacement magnitudes. These findings emphasize the interdependent roles of three key parameters: setback ratio (*b*/*D*), pile spacing ratio (*s*/*D*), and slope gradient (1 : *n*). Optimizing these factors collectively enhances structural stability and mitigates lateral deformation in pile groups.

### Lateral load required to induce 50 mm of pile head displacement

**Figure 6:**
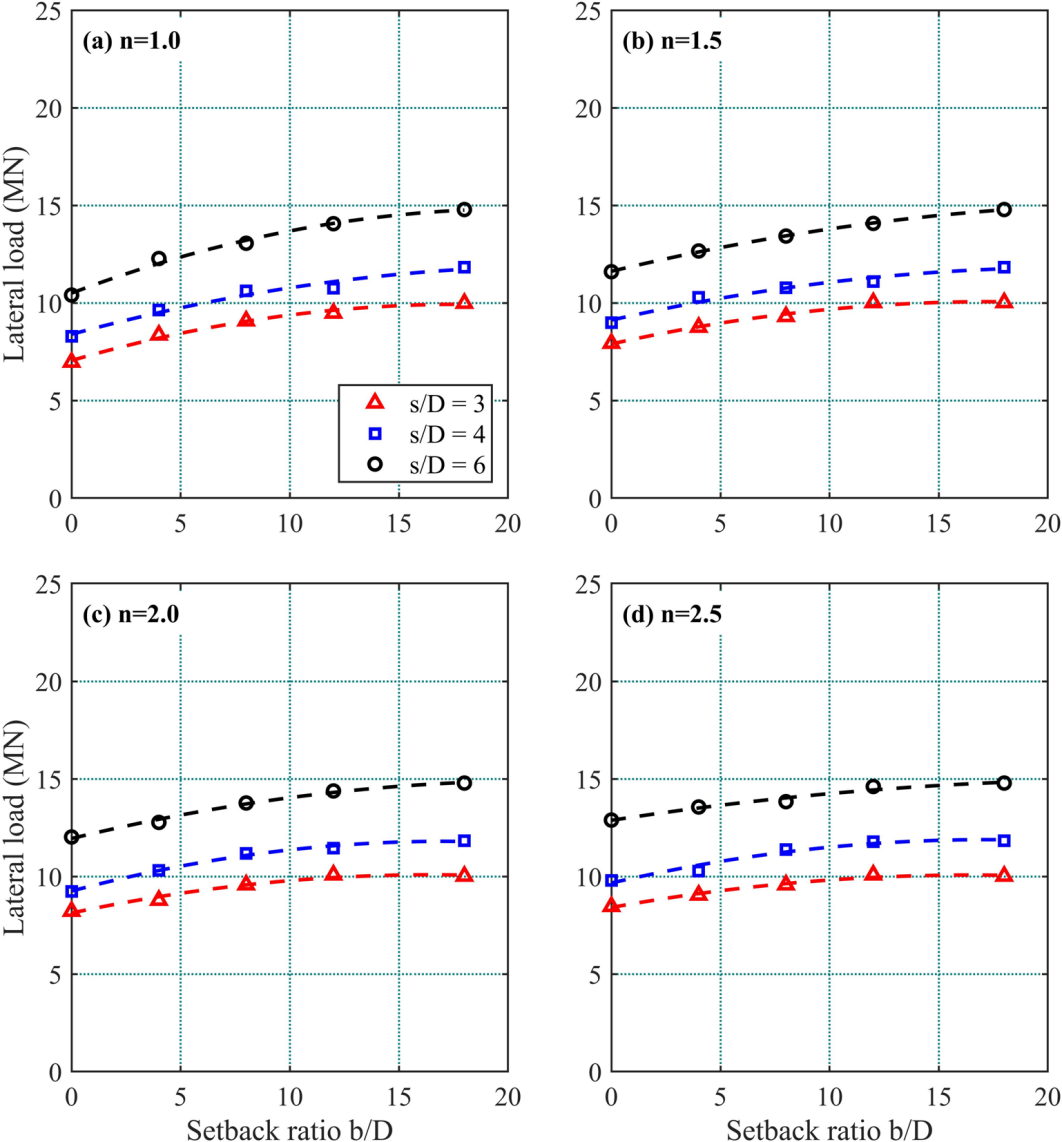
Lateral load required to induce a 50 mm of pile head lateral displacement in a 1*V* : *nH* slope: (**a**) $n=1.0$; (**b**) $n=1.5$; (**c**) $n=2.0$; and (**d**) $n=2.5$.

Table 2 and Fig. 6 presents the lateral load required to induce 50 mm of pile head displacement in front pile $F_1$ as a function of setback ratio *b*/*D*, analyzed for four slope gradients (1 : *n*). The influence of pile spacing ratio *s*/*D* is systematically examined in Fig. 6a–d. Across all configurations, the lateral load increases with *b*/*D*, irrespective of *s*/*D* or slope gradient. This demonstrates that higher setback ratios enhance lateral load capacity – greater *b*/*D* values necessitate increased lateral forces to achieve the target displacement. All subfigures exhibit a consistent upward trend, demonstrating a direct proportionality between setback ratio (*b*/*D*) and lateral resistance. This highlights *b*/*D* as a key design parameter for enhancing pile performance under lateral loads.

**Table 2:** Lateral load (MN) required to induce 50 mm of pile head displacement.

Setback	Lateral load induced under various slope gradients and pile spacings											
ratio	1 : 1			1 : 1.5			1 : 2			1 : 2.5		
$\varvec{b/D}$	*S*/*D*=3	*S*/*D*=4	*S*/*D*=6	*S*/*D*=3	*S*/*D*=4	*S*/*D*=6	*S*/*D*=3	*S*/*D*=4	*S*/*D*=6	*S*/*D*=3	*S*/*D*=4	*S*/*D*=6
0	7.0	8.3	10.4	7.9	9.0	11.6	8.2	9.2	12.0	8.5	9.8	12.9
4	8.4	9.6	12.3	8.8	10.3	12.7	8.8	10.3	12.8	9.0	10.3	13.6
8	9.1	10.6	13.1	9.3	10.8	13.4	9.6	11.2	13.8	9.6	11.4	13.8
12	9.5	10.8	14.1	10.0	11.1	14.1	10.1	11.5	14.4	10.1	11.8	14.6
18	10.0	11.8	14.8	10.0	11.8	14.8	10.0	11.8	14.8	10.0	11.8	14.8

For the 1:1 slope (Table 2 and Fig. 6a), the lateral load required to induce 50 mm displacement increases from 7.0 MN at $b/D=0$ (pile at slope crest) to 9.5 MN at $b/D=12$ for $s/D=3$. Compared to the 10 MN load required in level ground, the $b/D=0$ case achieves only 70% of this capacity. At $b/D=12$, the lateral load reaches 95% of the level-ground value, indicating near-recovery of full capacity.

For slopes of 1 : 1.5, 1 : 2.0,  and 1 : 2.5,  pile capacity similarly increases with higher *b*/*D* ratios, though the rate of improvement diminishes for $b/D>12$. The study identifies $b/D=12$ as the critical setback distance beyond which pile group behavior converges to level-ground performance. The most significant capacity reductions are observed at $b/D=0$, where limited soil mobilization near the slope crest significantly reduces lateral resistance. These trends highlight that the influence of *b*/*D* weakens in gentler slopes, while steeper slopes remain more responsive to setback ratio changes.

For all slope gradients (1 : *n*), pile groups with higher pile spacing ratios (*s*/*D*) demand greater lateral loads to achieve 50 mm displacement. For instance, inducing 50 mm displacement required 16% and 47% higher loads for $s/D=4$ and $s/D=6$, respectively, compared to $s/D=3$. This confirms that larger *s*/*D* ratios improve lateral load capacity at any given *b*/*D*.

These results align with Ayasrah’s findings^31^ where increased *s*/*D* ratios reduced group interaction effects and improved pile group performance. The same principle extends to strip footing behavior^32^, underscoring its broader applicability in geotechnical systems.

### Bending moments in pile

**Figure 7:**
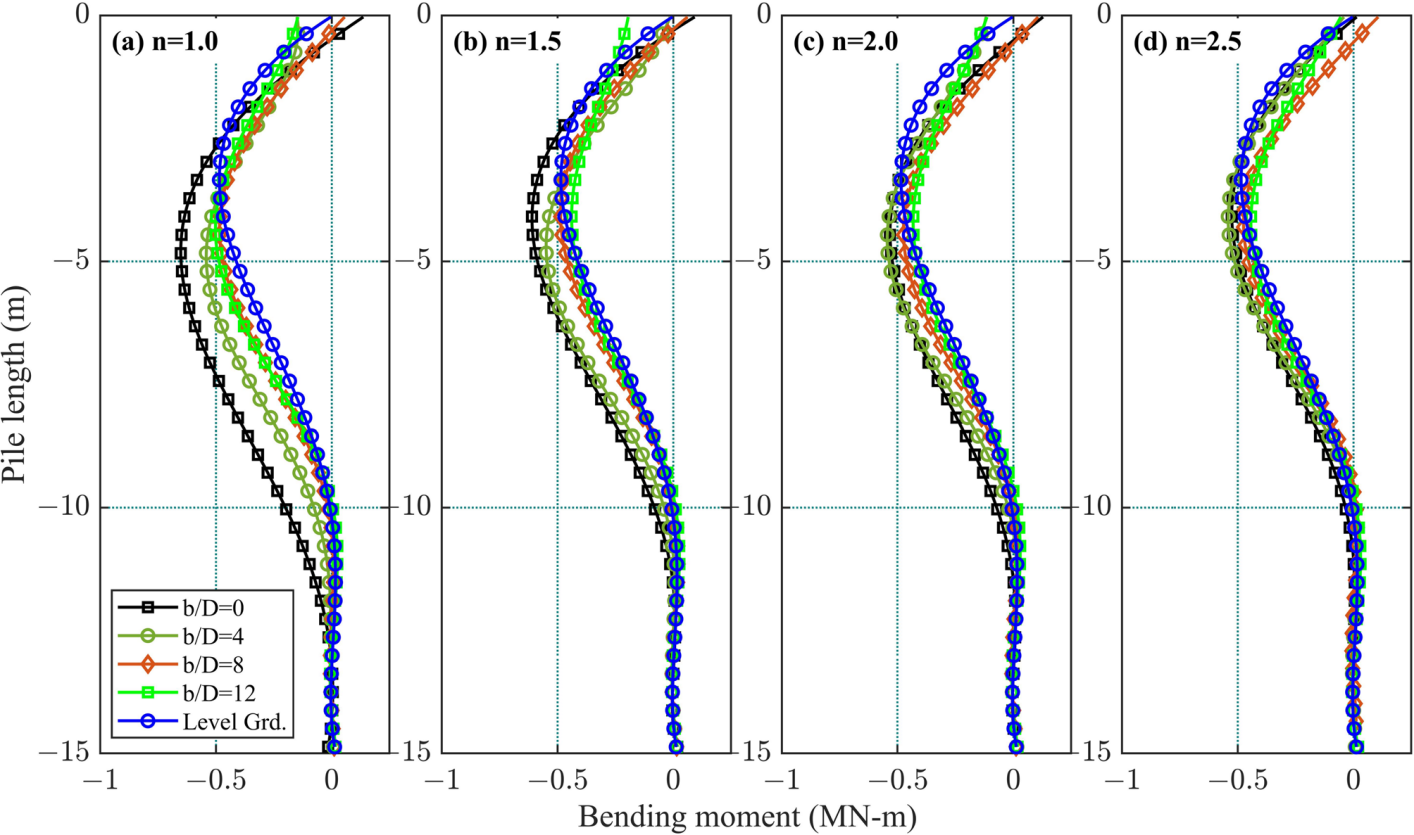
Typical bending moment profiles for $s/D=3$ in a 1*V* : *nH* slope: (**a**) $n=1.0$; (**b**) $n=1.5$; (**c**) $n=2.0$; and (**d**) $n=2.5$.

Figure 7 presents the bending moment distribution along the depth (*z*-axis) of front pile $F_1$ under a 15 MN lateral load, analyzed for setback ratios $b/D=0, 4, 8,$ and 12 with a fixed spacing ratio $s/D=3$. Fifth-degree polynomial curve fits were applied to the pile deflection profiles in Fig. 4 using the displacement function *y*(*z*) defined in Eq. (2). Bending moments *M*(*z*) at depth *z* were then calculated using the pile’s flexural rigidity $E_p I_p$ and the second derivative of *y*(*z*) as per Eq. (3).

**Table 3:** Maximum bending moment in the front pile $F_1$ and the normalized depth (*z*/*D*) of the maximum bending moment, at various *b*/*D* ratios (for $s/D = 3$ only).

Setback ratio *b*/*D*	Maximum bending moment in various slopes							
	1 : 1		1 : 1.5		1 : 2		1 : 2.5	
	BM (kN-m)	*z*/*D*	BM (kN-m)	*z*/*D*	BM (kN-m)	*z*/*D*	BM (kN-m)	*z*/*D*
0	652	9.6	610	8.5	533	8.7	525	8.2
4	542	9.8	549	9.2	544	9.0	540	8.3
8	485	8.8	486	8.3	475	8.7	475	8.5
12	500	8.7	439	8.3	433	8.4	443	8.4
Level Grd.	485	6.7	485	6.7	485	6.7	485	6.7

Figure 7 demonstrates that bending moment profiles exhibit the largest deviations across all setback ratios (*b*/*D*) in the steepest 1 : 1 slope, with deviations progressively diminishing in gentler slopes, reaching minimal differences in the 1 : 2.5 slope case. Furthermore, bending moment profiles maintain consistent shapes across all *b*/*D* ratios, indicating that *b*/*D* primarily modulates magnitude scaling rather than distribution patterns. This confirms that setback ratios influence the intensity of bending moments but preserve their fundamental depth-dependent behavior, regardless of slope gradient.

The analysis reveals a consistent trend across slope gradients (1 : *n*): smaller setback ratios (*b*/*D*) correlate with higher maximum bending moments, as summarized in Table 3. For instance, in the slopes of 1 : 1 and 1 : 1.5, the highest maximum bending moments–652 kN-m and 610 kN-m, respectively–occurred at $b/D=0$. In the slopes of 1 : 2 and 1 : 2.5, marginally higher maximum bending moments (544 kN-m and 540 kN-m) were recorded at $b/D=4$. However, the difference between $b/D=0$ and $b/D=4$ cases remains below 3%, a discrepancy potentially attributable to mesh discretization uncertainties.

Level ground conditions exhibit the smallest bending moments, contrasting sharply with piles near slopes. This confirms that piles closest to the slope crest (smallest *b*/*D*) develop the highest bending moments, emphasizing the critical role of setback distance in mitigating structural demands.

The study observed that maximum bending moments in piles near slopes typically occur at depths between 8*D* and 10*D* (Table 3), whereas piles on level ground exhibit the lowest bending moment at a shallower depth of 6.7*D*. The depth of maximum shearing resistance varies with slope gradient, with steeper slopes shifting the critical depth of bending moments and shear forces. Yin et al.^33^ attributed this shift to reduced soil mobilization efficiency in steeper slopes, where altered stress distributions hinder full shearing resistance development.

Yin et al.^33^, who conducted laboratory model tests on laterally loaded single piles in sand slopes, reporting increased lateral deflection, bending moment, and shear force with steeper slopes. Their study found maximum bending moments at depths of 1.6*D* (level ground), 3.6*D* (30$^\circ$ slope; 1 : 1.7), and 5.6*D* (60$^\circ$ slope), demonstrating a direct correlation between slope steepness and critical bending moment depth.

The deeper bending moment depths observed in this study ($8D-10D$) compared to the $1.6D-5.6D$ range reported by^33^ stem from scaling disparities inherent to their experimental setup. Critical differences include orders-of-magnitude variations in lateral load magnitude (50 N vs. 15 MN), stress field conditions (model pile embedment of 900 mm vs. full-scale 15 m), and load application height (400 mm vs. 0.5 m above ground). These scaling factors–particularly the reduced load intensity and geometric similitude limitations in the laboratory tests–explain the shallower bending moment depths in^33^. In contrast, full-scale field conditions in this study better represent stress redistribution mechanisms that shift critical depths downward.

## Discussion

Lateral loads induce bending moments in piles, triggering pile cap rotation and lateral deflection that activate soil-structure interaction and mobilize shear resistance^4,33^. Abbas et al.^8^ highlights the influence of pile head conditions: fixed-head piles (rigidly connected to caps) exhibit superior lateral resistance compared to free-head configurations. Zhang et al.^34^ further demonstrates that increased axial loads enhance lateral resistance in prefabricated shear walls but compromise structural ductility under seismic conditions.

This study observed significant reductions in bending moments for front pile $F_1$ as slope gradients increased (1 : 1 to 1 : 2.5), likely due to diminished lateral soil support in steeper slopes when piles near the slope crest displaced toward the sloping side. These findings underscore the need for simplified methods to estimate lateral soil resistance in geometrically complex scenarios. A promising approach, originally developed for ground berm design^35^, is evaluated here for adaptation to pile-slope systems.

**Figure 8:**
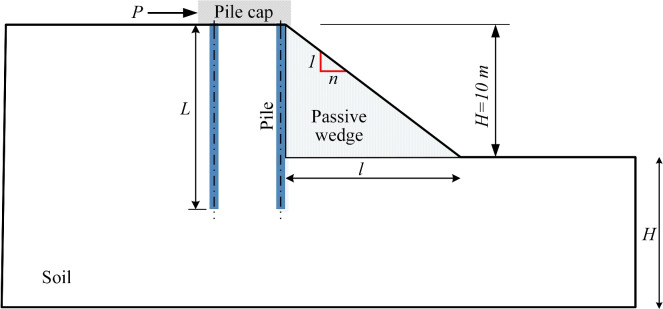
Passive soil wedge in front of the pile group.

For level ground conditions, laterally loaded piles mobilize passive resistance in the adjacent soil. While this resistance decreases with steeper slope gradients, it remains a critical component of the pile group’s lateral capacity^4,7^. Engineers commonly calculate the passive resistance per unit length ($P_p$, in kN/m) for level ground using Bell’s equation^36^, a modification of Rankine’s earth pressure theory incorporating cohesion:

4 $$\begin{aligned} P_p = \frac{1}{2} \gamma H^2 K_p + 2c'H\sqrt{K_p} \end{aligned}$$

where $\gamma$ is the soil unit weight; *H* is the height of the soil wedge (Fig. 8); $c'$ is the apparent cohesion; and $K_p$ is the coefficient of passive lateral earth pressure, which is given by

5 $$\begin{aligned} K_p = \tan ^2\left( 45^o + \frac{\phi '}{2} \right) \end{aligned}$$

The calculation assumes lateral earth pressure contributions from individual piles while disregarding group effects. As lateral loads intensify, overlapping shear zones between adjacent piles amplify shear resistance beyond capacities achievable by isolated piles. This interaction mechanism proves critical for optimizing lateral load performance^10,21,37^.

In sloping ground conditions, the slope face opposing pile movement functions analogously to a soil berm in retaining systems (Fig. 8). Similar to constructed berms–which improve stability and reduce deflection in embedded retaining structures through base sliding resistance^35,38^–a $c'-\phi '$ slope contributes sliding resistance via its self-weight and soil shear strength. This resistance counters active earth pressures, mitigating structural sliding or failure.

Design codes such as Eurocode 7^39^ and the LRFD Bridge Design Specifications^40^ explicitly mandate incorporating sliding resistance in retaining structure analysis. For pile groups near slopes, this principle implies that the slope’s inherent sliding capacity–governed by geometry (1 : *n*) and setback ratio (*b*/*D*)–critically influences lateral load distribution and deflection control.

The sliding resistance $R_s$ of a berm (width *l*) can be calculated by modeling it as a rigid body sliding along a potential failure plane, with the Mohr-Coulomb failure criterion applied to its base^38,39^:

6 $$\begin{aligned} R_s = c'l + \frac{1}{2}\gamma H l \tan \phi ' = c'l + W \tan \phi ' \qquad \text {(kN per m run)} \end{aligned}$$

where *W* is the self-weight of the berm.

The equivalent coefficient of passive earth pressure $K'_p$, which incorporates sliding resistance, provides a simplified representation of the berm’s contribution through an adjusted passive pressure coefficient for design. Employing the equivalent fluid pressure concept^41,42^, $K'_p$ is derived by equating the berm’s sliding resistance to the passive earth pressure force acting on level ground. This approach yields $K'_p$ values consistently lower than the conventional passive earth pressure coefficient $K_p$ calculated via Eq. (5).

7 $$\begin{aligned} K'_p = \frac{R_s}{\frac{1}{2}\gamma H^2} \quad < \quad K_p \end{aligned}$$

**Table 4:** Sliding resistance in slope with different gradients.

Slope gradient	Base width *l* (m)	Area of soil wedge (m^2^)	Weight of soil wedge *W* (kN)	Frictional force ${WD\tan \phi '}$ (kN)	Cohesion force ${c'LD}$ (kN)	Sliding resistance ${R_s}$ (kN)
1 : 1.0	10	50	1000	289	250	539
1 : 1.5	15	75	1500	433	375	808
1 : 2.0	20	100	2000	577	500	1077
1 : 2.5	25	125	2500	722	625	1347

Table 4 presents sliding resistance values for a 0.5 m diameter pile without explicit consideration of spacing ratio *s*/*D*. The sliding resistance in slopes or soil wedges (Fig. 8) varied significantly with gradient, increasing as slopes gentled due to greater passive soil wedge mass. This mechanism explains the higher pile displacements and bending moments observed in steeper slopes (e.g., 1 : 1) compared to gentler ones (e.g., 1 : 2.5).

Assuming independent sliding resistance per pile (neglecting group effects), total soil resistance for four piles reached 2.2 MN (1 : 1 slope) and 5.4 MN (1 : 2.5 slope). However, the applied lateral load of 15 MN exceeded these values, necessitating additional resistance from the concrete piles. The steeper 1 : 1 slope exhibited a larger deficit between sliding resistance and applied load, resulting in proportionally greater pile displacements and bending moments than the 1 : 2.5 slope.

For level ground conditions (ignoring group effects), Eqs. (5) and (4) yield a passive resistance $P_p=2366$ kN for a 0.5 m diameter pile. This value–derived without slope geometry considerations–exceeds the sliding resistance of the 1 : 1 slope ($R_s=539$ kN) by a factor of 4.39 and the 1 : 2.5 slope ($R_s=1347$ kN) by 1.76 times, thereby overestimating the soil slope’s lateral resistance.

In the sliding resistance concept, the soil wedge in front of the rear piles differs from the front piles and is influenced by the spacing between piles and the presence of the front piles. Unlike the front piles, whose soil wedges can displace more freely, the rear piles experience interference from the front piles, which limits movement and provides additional lateral resistance. This effect becomes more significant on sloped ground, where front piles engage more available soil resistance, and rear piles interact with already disturbed soil. This study assumes uniform lateral resistance for all piles to create a simplified baseline for comparing different configurations, focusing on how slope gradient influences behavior. Although this does not capture the full complexity of pile-soil interaction, it helps clarify the relative performance of each setup. Future studies may better represent these interactions by incorporating more detailed models, such as 3D nonlinear analyses or varying resistance assignments per pile.

## Conclusions

This study addresses critical gaps in understanding the lateral response of pile groups near slopes through 3D finite element analysis (COMSOL) of $2 \times 2$ pile groups in cohesive-frictional ($c'-\phi '$) soils. The analysis systematically evaluates three pile spacing ratios ($s/D=3, 4, 6$), four slope gradients (1 : 1.0, 1 : 1.5, 1 : 2.0, 1 : 2.5), and four setback ratios ($b/D=0, 4, 8, 12$) to quantify their interdependent effects on lateral capacity, displacement, deflection profiles, and bending moments. A novel aspect of the research is its adaptation of passive soil wedge and sliding resistance concepts–commonly used in berm-supported retaining wall design–explain how slope geometry contributes to lateral resistance. Key findings include:

- Pile setback distance (*b*/*D*) is the most critical factor affecting lateral capacity. Setbacks less than $b/D = 12$ significantly reduce performance, while further increases beyond this point yield diminishing improvements. Piles at $b/D=0$ (slope crest) exhibit $15-35$% lower capacity than those at $b/D=12$.
- Pile spacing (*s*/*D*) enhances lateral capacity. Wider spacing reduces group interaction, requiring up to 1.47 times more load to induce the same displacement and resulting in $28-58$% lower deflection under a given load than in tighter configurations. Pile spacing ($s/D=6$) enhances lateral capacity by up to 50% compared to $s/D=3$.
- Steeper slopes (1 : 1) significantly degrade capacity due to reduced passive resistance, increasing deflection and bending moments by $20-45$% relative to gentler slopes (1 : 2.5) or level ground. Sliding resistance in 1 : 1 slopes drops to 40% of that in 1 : 2.5 slopes, transferring greater load to piles.
- Adapting passive wedge and sliding resistance concepts from berm-supported retaining walls clarifies how slope geometry contributes to lateral resistance and explains how slope geometry governs lateral resistance, bridging geotechnical design principles with pile group behavior, offering practical design insights.
- While numerical models have limitations, the findings provide essential guidance for safer slope-adjacent pile group design.
- Future work should integrate AI-based methods to address heterogeneous soil layers and refine load distribution predictions.

By integrating slope geometry, soil mechanics, and group interaction effects, these findings offer new insights and practical guidelines for safer, more efficient foundation design on sloped terrain.

## Data Availability

All data generated or analysed during this study are included in this published article.

## References

[1] Deendayal, R., Muthukkumaran, K. & Sitharam, T. G. Analysis of laterally loaded group of piles located on sloping ground. *Int. J. Geotech. Eng.* **14**(5), 580–588. 10.1080/19386362.2018.1448521 (2020).

[2] Elhakim, A. F., El Khouly, M. & Awad, R. Three-dimensional modeling of laterally loaded pile groups resting in sand. *HBRC J.* **12**(1), 78–87. 10.1016/j.hbrcj.2014.08.002 (2016).

[3] Zhang, J., Wang, X., Wang, H. & Qin, H. Model test and numerical simulation of single pile response under combined loading in slope. *Appl. Sci.* **10**(17), 6140. 10.3390/app10176140 (2020).

[4] Sivapriya, S. V. & Ramanathan, R. Load-displacement behavior of a pile on a sloping ground for various l/d ratios. *Slovak J. Civ. Eng.* **27**(1), 1–6. 10.2478/sjce-2019-0001 (2019).

[5] Muthukkumaran, K. & Almas Begum, N. Experimental investigation of single model pile subjected to lateral load in sloping ground. *Geotech. Geol. Eng.* **33**, 935–946. 10.1007/s10706-015-9875-7 (2015).

[6] Khati, B. S. & Sawant, V. A. Comparison of lateral response of pile group in series and parallel arrangement near sloping ground. *Int. J. Geotech. Eng.* **14**(6), 686–695. 10.1080/19386362.2019.1617478 (2020).

[7] Khati, B. S. & Sawant, V. A. Experimental study of laterally loaded pile group in square arrangement near sloping ground. *Int. J. Geomech., ASCE* **21**(2), 04020257. 10.1061/(ASCE)GM.1943-5622.0001911 (2021).

[8] Abbas, S. A., Al-Rekabi, W. S. & Al-Aboodi, A. H. Analysis of laterally loaded (22) square pile groups using finite element method. * Proc. of Sixth Int. Conf. On Advances in Civil and Structural Engineering–CSE 2016*** 0**, 7–12, https://theired.org/conference/paper/analysis-of-laterally-loaded-22-square-pile-groups-using-finite-element-method-2975 (2016).

[9] Bahloul, K. M. Behavior of laterally loaded pile groups adjacent to a slope - a numerical study. *J. Eng. Res. Egypt* **6**(5), 155–160 (2022) https://digitalcommons.aaru.edu.jo/erjeng/vol6/iss5/17.

[10] Sivapriya, S. V. & Gandhi, S. R. Evaluating the lateral capacity of flexible pile group subjected to lateral load in sloping ground. *Res. Square Preprint* **0**, 1. 10.21203/rs.3.rs-4810996/v1 (2024).

[11] Alinejad, R. M., Bayat, M., Nadi, B. & Pakbaz, M. S. Experimental study of axially loaded pile group near a sloping ground. *Period. Polytech. Civ. Eng.* **67**(2), 382–391. 10.3311/PPci.18334 (2023).

[12] Navale, A. V., Solanki, C. H. & Sawant, V. A. Lateral response of 22 pile group embedded in cohesive soil near slope. *U. Porto J. Eng.* **9**(4), 1–19. 10.24840/2183-6493_009-004_001761 (2023).

[13] Abu-Farsakh, M., Souri, A., Voyiadjis, G. & Rosti, F. Comparison of static lateral behavior of three pile group configurations using three-dimensional finite element modeling. *Can. Geotech. J.* **55**, 107–118. 10.1139/cgj-2017-0077 (2018).

[14] Fayyazi, M. S., Taiebat, M., Finn, W. D. L. & Ventura, C. E. Evaluation of p-multiplier method for performance-based design of pile groups. In * Proceedings of the 2nd International Conference on Performance-Based Design in Earthquake Geotechnical Engineering (PBD-II)* (2012). Paper No. 11.05.

[15] Khatibi, S. K. Experimental comparison of shadowing effect and edge effect in pile group of integral bridge. *Arab. J. Geosci.* **14**, 1218. 10.1007/s12517-021-07615-0 (2021).

[16] McCormac, J. C. & Brown, R. H. * Design of Reinforced Concrete (ACI 318-11 Code Edition)* (John Wiley & Sons, Hoboken, NJ, 2014), 9th edn.

[17] Randolph, M. F. Design methods for pile groups and piled rafts. In * XIII International Conference on Soil Mechanics and Foundation Engineering*, vol. 5, 61–82 (New Delhi, India, 1994). State of the Art Report.

[18] Horikoshi, K. & Randolph, M. F. A contribution to the design of piled rafts. *Géotechnique* **48**, 301–313. 10.1680/geot.1998.48.3.301 (1998).

[19] Mandolini, A., Russo, F. & Viggiani, C. Rational design of piled raft. In * Proceedings of the 11th International Conference on Modern Building Materials, Structures and Techniques (MBMST 2013)*, vol. 57, 45–52 ( Vilnius Gediminas Technical University Press, Vilnius, Lithuania, 2013).

[20] Jamil, I. et al. Factors affecting the lateral contribution of a raft in a piled raft system. *Ain Shams Eng. J.* **14**, 101968. 10.1016/j.asej.2022.101968 (2023).

[21] Li, J. D., Zhang, Y. T., Yuan, G. Z. & Bian, T. Q. Numerical study on the lateral behavior of pile groups in toyoura sand using the hypoplastic constitutive model. *Sci. Rep.* **14**, 23364. 10.1038/s41598-024-74494-2 (2024). 39375482 10.1038/s41598-024-74494-2 PMC11458592

[22] COMSOL AB. * COMSOL Multiphysics User’s Guide*. Stockholm, Sweden, version 5.6 edn. (2021).

[23] Gui, M. W. & Alebachew, A. A. Responses of laterally loaded single piles subjected to various loading rates in a poroelastic soil. *Appl. Sci.* **12**(2), 617. 10.3390/app12020617 (2022).

[24] Winkler, E. * Die Lehre von der Elasticität und Festigkeit* ( Dominicus, Prague, 1867), 1st edn. Translated title: The Theory of Elasticity and Strength.

[25] Murthy, V. N. S. * Geotechnical Engineering: Principles and Practices of Soil Mechanics and Foundation Engineering* ( CRC Press, Boca Raton, FL, 2002).

[26] Huang, J. W. * Development of Modified p-y Curves for Winkler Analysis to Characterize the Lateral Load Behavior of a Single Pile Embedded in Improved Soft Clay*. Master’s thesis, Iowa State University (2011).

[27] Eberle, R. & Oberguggenberger, M. A new method for estimating the bending stiffness curve of non-uniform euler-bernoulli beams using static deflection data. *Appl. Math. Model.* **105**, 514–533. 10.1016/j.apm.2021.12.042 (2022).

[28] Ruesta, P. F. & Townsend, F. C. Evaluation of laterally loaded pile group at roosevelt bridge. *ASCE J. Geotech. Geoenviron. Eng.* **123**(12), 1153–1162. 10.1061/(ASCE)1090-0241(1997)123:12(1153) (1997).

[29] Rollins, K. P., Peterson, K. T. & Weaver, T. J. Lateral load behavior of full-scale pile group in clay. *ASCE J. Geotech. Geoenviron. Eng.* **124**(6), 468–478. 10.1061/(ASCE)1090-0241(1998)124:6(468) (1998).

[30] Rollins, K. M., Johnson, S. R., Petersen, K. T. & Weaver, T. J. Static and dynamic lateral load behavior of pile groups based on full-scale testing. In * Proceedings of the 13th International Offshore and Polar Engineering Conference* ( Honolulu, Hawaii, USA., 2003). Paper No. ISOPE-I-03-156.

[31] Ayasrah, M. Behavior of micropile (Type D) subjected to vertical load: Parametric numerical studies. *Appl. Mech.* **6**(4). 10.3390/applmech6010004 (2025).

[32] Ayasrah, M. & Fattah, M. Y. Finite element analysis of two nearby interfering strip footings embedded in saturated cohesive soils. *Civ. Eng. J.* **9**(3), 752–769. 10.28991/CEJ-2023-09-03-017 (2023).

[33] Yin, P. B., He, W. & Yang, J. Z. H. A simplified nonlinear method for a laterally loaded pile in sloping ground. *Adv. Civ. Eng.* 5438618. 10.1155/2018/5438618 (2018).

[34] Zhang, L. B. et al. Seismic behavior of cluster-connected prefabricated shear walls under different axial compression ratios. *Buildings* **14**, 2768. 10.3390/buildings14092768 (2024).

[35] Bashmakov, I. B. Analytical methods for calculating passive ground pressure in the construction of ground berms. In * Proc. 17th Asian Regional Conf on Soil Mechanics and Geotechnical Engineering: Smart Geotechnics for Smart Societies*, Zhussupbekov, Sarsembayeva & Kaliakin (Eds), CRC Press, London, 1009–1014, 10.1201/9781003299127-141 (2023).

[36] Bell, A. L. The lateral pressure and resistance of clay, and supporting power of clay foundations. *Min. Proc. Insti. Civ. Eng.* **199**, 233–272 (1915).

[37] Deendayal, R., Muthukkumaran, K. & Sitharam, T. G. Analysis of laterally loaded group of piles located on sloping ground. *Int. J. Geotech. Eng.* **14**(5), 580–588. 10.1080/19386362.2018.1448521 (2018).

[38] Smethurst, J. A. & Powrie, W. Effective-stress analysis of berm-supported retaining walls. *Proc. Inst. Civ. Eng. Geotech. Eng.* **161**(1), 39–48. 10.1680/geng.2008.161.1.39 (2008).

[39] European Committee for Standardization. * Eurocode 7: Geotechnical design - Part 1: General rules*. With National Annex (2004).

[40] American Association of State Highway and Transportation Officials. *AASHTO LRFD Bridge Design Specifications* (American Association of State Highway and Transportation Officials, Washington, D.C., 2020).

[41] American Association of State Highway and Transportation Officials. *Standard Specifications for Highway Bridges* 17th edn. (Washington, D.C., 2002).

[42] Coduto, D. P. *Foundation Design: Principles and Practices* ( Prentice Hall, Upper Saddle River, NJ, 2001), 2nd edn.

